# Numerical Magnitude Affects Temporal Memories but Not Time Encoding

**DOI:** 10.1371/journal.pone.0083159

**Published:** 2014-01-29

**Authors:** Zhenguang G. Cai, Ruiming Wang

**Affiliations:** 1 School of Psychology, University of Plymouth, Plymouth, United Kingdom; 2 Center for Studies of Psychological Application, School of Psychology, South China Normal University, Guangzhou, China; CEA.DSV.I2BM.NeuroSpin, France

## Abstract

Previous research has suggested that the perception of time is influenced by concurrent magnitude information (e.g., numerical magnitude in digits, spatial distance), but the locus of the effect is unclear, with some findings suggesting that concurrent magnitudes such as space affect temporal memories and others suggesting that numerical magnitudes in digits affect the clock speed during time encoding. The current paper reports 6 experiments in which participants perceived a stimulus duration and then reproduced it. We showed that though a digit of a large magnitude (e.g., 9), relative to a digit of a small magnitude (e.g., 2), led to a longer reproduced duration when the digits were presented during the perception of the stimulus duration, such a magnitude effect disappeared when the digits were presented during the reproduction of the stimulus duration. These findings disconfirm the account that large numerical magnitudes accelerate the speed of an internal clock during time encoding, as such an account incorrectly predicts that a large numerical magnitude should lead to a shorter reproduced duration when presented during reproduction. Instead, the findings suggest that numerical magnitudes, like other magnitudes such as space, affect temporal memories when numerical magnitudes and temporal durations are concurrently held in memory. Under this account, concurrent numerical magnitudes have the chance to influence the memory of the perceived duration when they are presented during perception but not when they are presented at the reproduction stage.

## Introduction

In physics, time is an integrated dimension of the physical world; similarly, in our daily life, perceived time is one of the many dimensions of a perceived event (e.g., the duration, together with the pitch and loudness, of a tone). Much recent research has shown that the perceived duration of an event is influenced by other concurrent dimensions of the event. Xuan, Zhang, He, and Chen [1] demonstrated that people judge a stimulus to be lasting for longer if the stimulus consists of more dots, is larger in size, has greater luminance, or is a digit of a larger magnitude. Other studies have corroborated these findings. For example, the perceived duration of a digit (e.g., 2 or 9) is longer for a larger magnitude digit [2], [3]. Similarly, perceived duration increases as a function of concurrent numerosity (e.g., quantity of dots in the stimulus) [4], [5]. Subjective time also varies as a function of concurrent spatial information, with time being perceived as longer when it is concurrently accompanied by a larger volume of space [6], a longer line on the screen [7], or a gesture covering a longer distance [8]. These demonstrations have led researchers to propose that that time, together with other quantifiable physical dimensions such as numerosity, luminance and space, is processed as mental magnitudes [9]–[11]. As a mental magnitude is an approximate (and hence noisy) representation of the quantity information extracted from a physical dimension, it is thus possible for concurrent non-temporal magnitudes (e.g., of space and numerosity) to bias temporal magnitudes [9]. The magnitude account also explains why concurrent duration can sometimes bias the perception of spatial distance [12] and numerosity [4].

**Figure 1:**
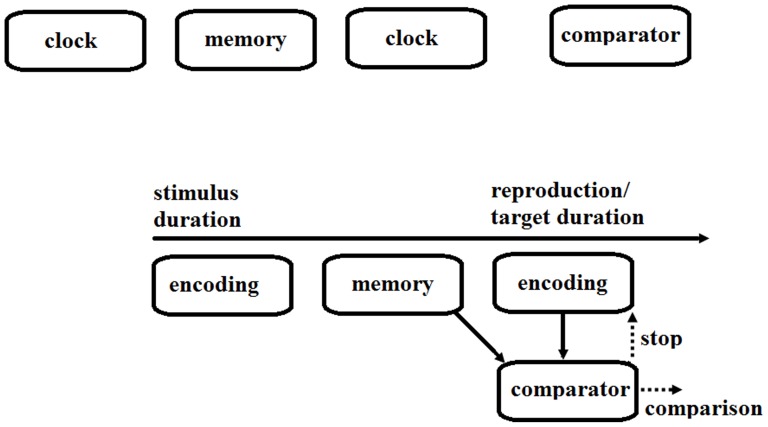
Cognitive processes involved in time perception. First, a stimulus duration is encoded and then kept in memory. Next, the comparator retrieves the remembered duration with which the newly encoded duration (i.e., the duration being reproduced in a reproduction task or a target duration in a comparison task) is compared. The comparator stops the reproduction when the reproduced duration is similar enough to the remembered duration, or makes a comparison judgment based on the relative amounts of time between the remembered duration and the target duration in a comparison task.

However, it is less clear when and where during time perception non-temporal magnitude information interacts with temporal durations. In time perception, people need to first encode a stimulus duration, hold it in memory, and later retrieve the temporal memory for temporal judgment (e.g., time reproduction or comparison) [13], [14]; see [15], [16] for recent reviews. For example, according to the internal clock model [13], [14], temporal durations are encoded by a clock-like device, which registers time with a variable speed (the faster the clock speed, the more subjective time is perceived). The perceived duration is then stored in memory and later retrieved for time judgment (e.g., to stop a reproduction when the reproduced duration is subjectively equivalent to the remembered duration, or to decide whether a perceived target duration is longer or shorter than a previously remembered stimulus duration). Figure 1 illustrates the cognitive processes involved in time perception. Under this analysis, non-temporal magnitude information may affect the clock speed during time encoding (e.g., a larger magnitude accelerates the clock speed, hence larger amount of time being perceived) or the temporal memory of a perceived duration (e.g., a larger non-temporal magnitude, relative to a smaller one, lengthens the temporal memory).

Research has shown that manipulations of clock speed during time encoding and temporal memories result in different behavioral effects. Meck and Church [17] showed that, while the intake of protein and carbohydrate respectively increased and decreased the clock speed in rats, leading to an immediate effect of shortened and lengthened waiting time for food, the intake of choline affected temporal memories, leading to a gradual shift towards shortened waiting time (see also [18], [19] for similar findings). Behavioural studies in humans also show that external manipulations can change perceived durations by altering the clock speed. Rapid repetitive stimulation such as auditory click trains and visual flickers have been found to increase the clock speed during time encoding. It was shown that a duration was perceived 10% longer if it immediately followed a five-second click train at 5 Hz or 25 Hz, suggesting that the click train speeded up time encoding rate [20], [21]. When judging which of two previously memorized reference durations a target duration was more similar to, participants more often chose the shorter reference duration when the reference durations were presented concurrently with visual flicker, but more often chose the longer reference duration when the target duration itself was accompanied with visual flicker [22]. These stage-dependent reverse effects on timing judgments reflect an acceleration in the clock speed during time encoding in the presence of visual flicker, hence longer perceived time for whichever duration that was perceived with concurrent visual flicker. Therefore, it is possible that concurrent magnitudes influence the speed in which a stimulus duration is being encoded, hence biasing the subjective time that is perceived.

Alternatively, concurrent magnitudes may bias the memory of a stimulus duration. The representation of a duration is susceptible to interference while being held in memory. The most well-known effect of memory on time is probably the Vierordt effect, which refers to observations that, in reproducing or estimating a duration, people tend to be influenced by memories of previously perceived durations and as a result overestimate shorter durations but underestimate longer durations [23]–[26]. For instance, Jazayeri and Shadlen [25] had participants perceive stimulus durations of a particular range (e.g., 671–1023 ms, or 847–1200 ms). They found that within each range, participants over-reproduced shorter durations but under-reproduced longer durations; furthermore, a stimulus duration (e.g., 900 ms) was reproduced longer when it belonged to the shorter end of a larger range (e.g., 847–1200 ms) than when it belonged to the longer end of a smaller range (e.g., 671–1023 ms). These results strongly suggest that the reproduction of a just-perceived duration can be influenced by previously perceived/reproduced durations. Similarly, when participants have concurrently held in memory two reference durations with which target durations are compared, they tend to overestimate the shorter reference duration and underestimate the longer reference duration [27], [28]. This occurs supposedly because the memories of the two reference durations pull on each other while both are being held in memory. Thus, it is very likely that concurrent magnitude information from non-temporal dimensions such as space and digits can pull on the magnitude representation of a duration.

Therefore, it is possible that a magnitude may bias time by accelerating or slowing down the speed of time encoding, or by biasing the representation of a duration when it is being held in memory. The findings that the manipulation of the clock speed using external stimuli result in stage-dependent reverse effects [22] offer an opportunity for us to determine the locus of the magnitude effect by examining how the effect changes depending on the stage at which a magnitude is presented. If magnitude information affects the clock speed during time encoding (during either time perception or reproduction), we should expect a stage-dependent reverse effects on timing behaviours. For instance, in a time reproduction task, when a large magnitude (relative to a small one) is presented at the perception stage (i.e., the stage during which participants perceive a stimulus duration), participants should accumulate more subjective time for the stimulus duration and accordingly reproduce a longer duration. When a large magnitude (relative to a small one) is instead presented at the reproduction stage (i.e., the stage during which participants produce a duration to match the memorized stimulus duration), participants should similarly accumulate more subjective time for the duration being reproduced and hence stop the reproduction earlier, resulting in a shorter reproduced duration. If magnitude information instead biases temporal memories, we should expect a different pattern of the magnitude effects. More specifically, concurrent magnitude should only affect time perception when it is presented at the perception stage, in which case the memory of the magnitude information can influence that memory of a duration, but not at the reproduction stage, in which case the memory of a duration will have been retrieved from the memory store and is therefore free from the influence of the magnitude information that is later presented during reproduction.

Recently, Cai and Connell (unpublished data) used the above rationale to determine whether spatial distance affects time encoding or temporal memories. In a time reproduction task, they manipulated visual flicker (a flickering or a static dot) and spatial distance (a long- or short-distance line) at either the time perception stage or the time reproduction stage. In the former setup, participants saw an experimental visual stimulus (a flickering/static dot or a long-/short-distance line) presented for a certain duration; then they held down the spacebar to reproduce that duration, during which a neutral visual stimulus (***) stayed onscreen. In the latter setup, a duration was presented using the neutral visual stimulus and participants saw one of the experimental visual stimuli while reproducing the duration. Visual flicker, which has been shown to affect clock speed [22], produced stage-dependent reverse effects, with the flickering dot leading to *longer* reproductions than the static dot when presented at the perception stage but *shorter* reproductions when presented at the reproduction stage. Spatial distance, however, produced an effect only when it was presented at the perception stage: the long-distance line, relative to the short-distance line, led to longer reproductions when presented at the perception stage but there was no effect of spatial distance presented at the reproduction stage. These findings suggest that though visual flicker affects the clock speed during time encoding [22], spatial distance does not. Instead, the findings suggest that spatial distance interacts with temporal durations when they are concurrently residing in memory: spatial distance has the opportunity to interfere with the memorized duration when presented at the perception stage, but not when presented at the reproduction stage (see Figure 1).

The above findings imply that the magnitude effect on time occurs at the time when a perceived duration is being held in memory concurrently with other magnitude information. However, a recent study seems to defy such a conclusion. Chang et al. [2] showed that though large magnitude digits (8 and 9) led to longer reproduced durations than small magnitude digits (1 and 2) when digits were presented at the perception stage, they led to *shorter* reproduced durations than small magnitude digits when digits were presented at the reproduction stage. Though they used these findings as evidence for common or closely connected representations between time and number (a conclusion we endorse), the stage-dependent reverse effects of numerical magnitudes on timing suggest that numerical magnitudes bias time encoding rather than temporal memories. The discrepancy between Cai and Connell (unpublished data) and Chang et al. [2] may reflect a difference in the nature of interaction between spatial distance and time on the one hand and between numerical magnitudes and time on the other. However, we noticed that the effect of numerical magnitudes presented during the reproduction stage was very small (though significant), with reproduced durations only 5 ms longer when accompanied by small magnitude digits than when accompanied by large magnitude digits. Thus, it is not sure whether the effect can be generalized or is simply a one-off accidental observation, probably due to participant sampling or data trimming (e.g., Chang et al. excluded reproductions shorter than 100 ms [1/3 of the shortest stimulus duration, 300 ms] or longer than 1000 ms [4/3 times the longest stimulus duration, 750 ms], a trimming method we believe might have resulted in excluding as outliers data that were otherwise normal reproduced durations).

Hence, the present study aimed to test whether the findings in Chang et al. can be replicated. Experiments 1 and 2 are replications of the two experiments in Chang et al. To preview, though we replicated the finding that large magnitude digits led to longer reproduced durations when they were presented at the perception stage, we did not observe any numerical magnitude effect when digits were presented at the reproduction stage. To test whether the lack of the numerical magnitude effect in Experiment 2 was due to inattention to the digit, in Experiment 3, we asked participants to reproduce the digit they saw during time reproduction. Still we failed to observe any numerical magnitude effect, suggesting that lack of numerical magnitude effect in Experiments 2 and 3 was not due to inattention to digits. Experiments 4a and 4b treated numerical magnitude as a continuous variable (1, 3, 5, 7, 9) rather categorical one. Again we observed the numerical magnitude effect at the perception stage but not at the reproduction stage. The null effect of numerical magnitude at the reproduction stage was further confirmed in Experiment 5, in which we increased the number of both participants and items, suggesting that the null effects are unlikely due to a lack of power in the experiments. Table 1 presents a summary of the manipulations in the experiments.

**Table 1:** Differences in experimental parameters among the experiments.

Experiment	Digit presentation	Magnitude manipulation	Digit reproduction
1	Perception	Categorical: 1/2 vs. 8/9	No
2	Reproduction	Categorical: 1/2 vs. 8/9	No
3	Reproduction	Categorical: 1/2 vs. 8/9	Yes
4a	Perception	Continuous: 1, 3, 5, 7, 9	No
4b	Reproduction	Continuous: 1, 3, 5, 7, 9	No
5	Reproduction	Continuous: 1, 3, 5, 7, 9	No

## Experiments 1 and 2

These experiments were replications of the two experiments in Chang et al. [2]. In Experiment 1, participants perceived the duration of a small magnitude digit (1 or 2) or a large magnitude digit (8 or 9) and then reproduced the duration; in Experiment 2, they perceived a stimulus duration, and reproduced the duration with either a small or large magnitude digit onscreen.

### Method

#### Ethics Statement

Ethic approval for these two experiments and the others reported in this paper was obtained from the Human Ethics Committee, Faculty of Science and Technology, University of Plymouth. Informed written consent was obtained from all participants according to the institutional guidelines of both the University of Plymouth and South China Normal University.

#### Participants

17 (15 females; mean age: 20.4) and 28 (25 females; mean age: 19.5) participants from the South China Normal University community were paid 10 Yuan (roughly $1.5) to respectively take part in Experiment 1 and Experiment 2. They all had normal or corrected-to-normal vision.

#### Materials

**Figure 2:**
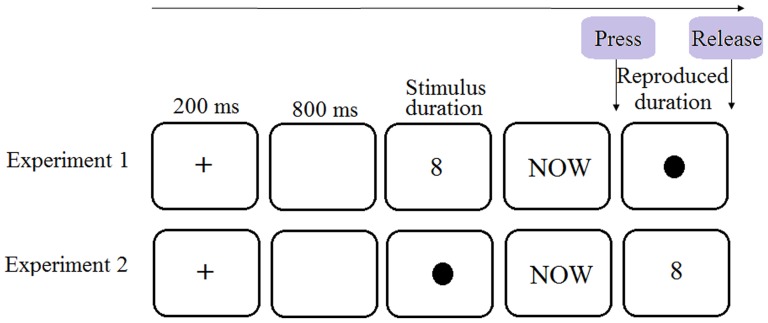
A schematic illustration of a trial in Experiments 1 and 2 (adapted from Chang et al. [2]). The dot was green in the experiments. Later experiments used the same or similar trial procedures. Experiment 3 differed from Experiment 2 only in having a 1000 ms blank interval between the offset of the green dot and the onset of “NOW” and having a digit reproduction task after time reproduction. Experiment 4a used the same trial procedure as in Experiment 1 and Experiment 4b and 5 used the same trial procedure as in Experiment 2.

The materials were the same as those used in Chang et al. [2]. In Experiment 1, Arabic digits of a small magnitude (1 or 2) or a large magnitude (8 or 9) were used to present one of the following stimulus durations: 300 ms, 450 ms, 600 ms, and 750 ms. Crossing the four digits and the four stimulus durations resulted in sixteen digit-duration combinations/trials, each occurring twice in a block of materials. The order of trials was randomized within a block. There were ten blocks, with a total of 320 trials. Experiment 2 was the same as Experiment 1 except that the digit was presented at the reproduction stage rather than the perception stage (see Figure 2).

#### Procedure

Participants were individually tested in a cubicle. The experiment was carried out in DMDX [29] on a Lenovo desktop with a 15 inch IBM monitor. After giving their written consent, participants had a practice session consisting of four trials before the main experiment. In Experiment 1, a trial began with a fixation cross lasting for 200 ms, followed by a blank screen of 800 ms. Then a digit was presented for one of the four stimulus durations. After the offset of the stimulus duration, the word “NOW” appeared on the screen as a cue for participants to start reproducing the stimulus duration by holding down the Key 0 on the number pad (a green dot appeared at the press of the key and remained onscreen for the whole reproduction). The procedure for Experiment 2 was the same except that the stimulus duration was presented with a green dot and a digit appeared and stayed onscreen during the reproduction. It should be noted that due to an oversight in script programming, our procedure differed from that of Chang et al. [2] in not having a 1000 ms blank interval between the offset of a stimulus duration and the onset of the cue word “NOW”. Figure 2 presents a schematic representation of the procedure. Participants could choose to take a break after each block.

#### Data analysis

As the trimming method adopted in Chang et al. [2] is likely to exclude data that were not outliers, we adopted a different trimming method and treated as outliers reproduced durations that were shorter than 1/4 of the stimulus duration or longer than 4 times the stimulus duration (the use of Chang et al.'s method did not change the findings though; see the result section). We adopted such a trimming method rather than one based on standard deviation because the latter method, due to the right-tailed distribution of time reproduction data, tends to include very brief reproductions caused by accidental key presses/releases (e.g., 2 or 3 *SD*s away from the mean would often result in a negative score). For the data analysis, we adopted linear mixed effects (LME) modeling [30] on the averaged reproduced duration per each combination of stimulus duration and numerical magnitude (e.g., 300 ms stimulus duration with the digit 9) per participant. LME modeling is a more appropriate method than ANOVA for our data because it enables us to treat both stimulus durations and numerical magnitude (when necessary) as continuous variables rather than categorical ones as in ANOVA analyses. It also allows for the consideration of all possible random effects such as individual differences in sensitivity to a particular manipulation (e.g., stimulus duration). Following the suggestion by Barr et al. [31], we used the maximal random effect structure (i.e., including all possible random slopes in addition to the random intercept) when possible in order to avoid otherwise inflated Type I error. When the maximal random effect structure model resulted in a convergence problem, we simplified the random effect structure by removing the correlations between the random intercept and the random slopes [31]. All fixed predictors were centered to reduce colinearity. We reported unstandardized coefficients for the fixed effects and the associated *t*-test. The analyses were conducted in R (version 2.10.1). As the R package we used for LME modeling, lme4, does not provide degrees of freedom and hence no *p*-values for *t*-tests, *p*-values were computed using Monte Carlo Markov Chain simulation.

### Results and discussion

**Figure 3:**
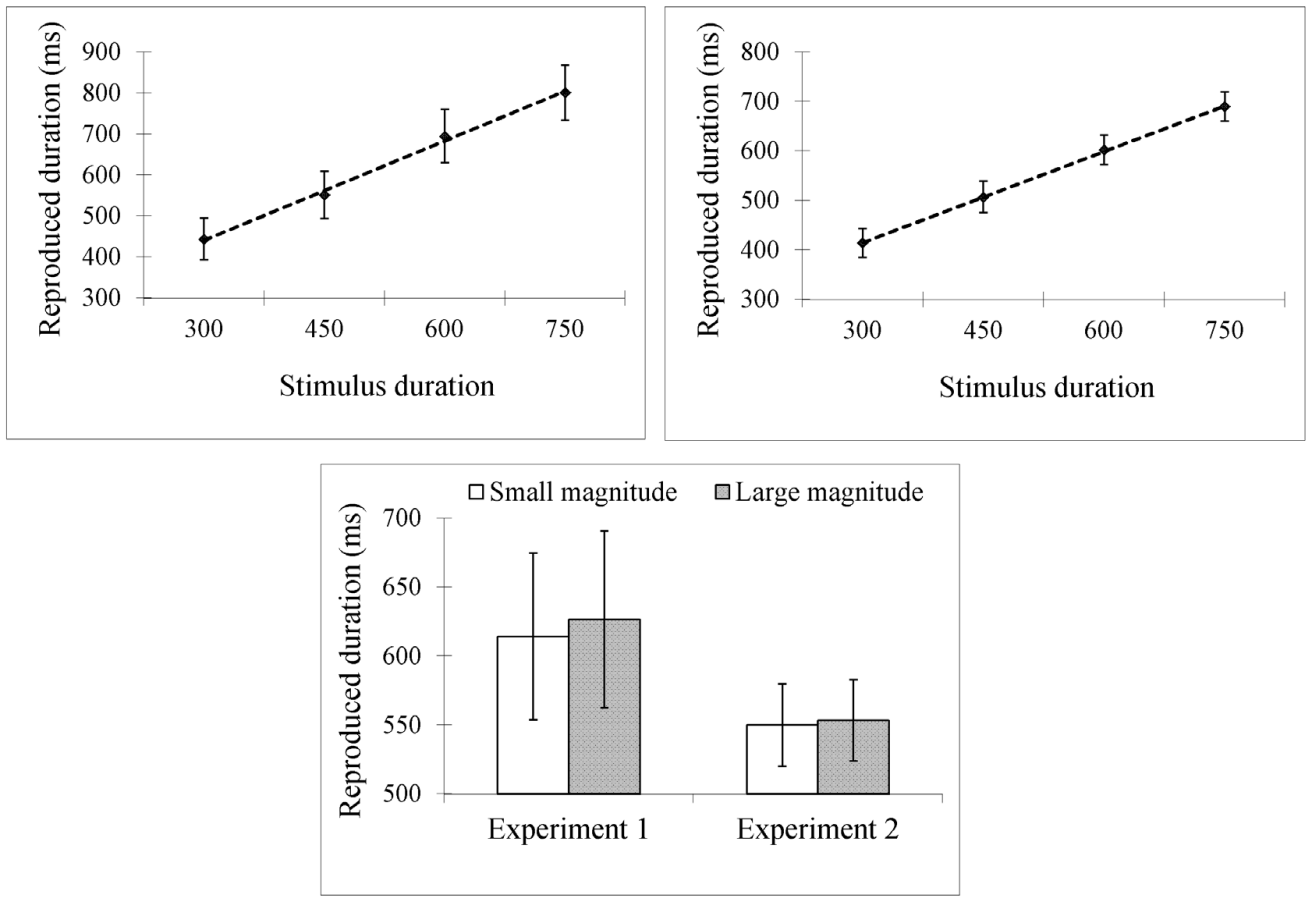
Results for Experiments 1 and 2. In Experiment 1, reproduced durations increased as a function of stimulus duration (A) and numerical magnitude (C, left); in Experiment 2 reproduced durations increased as a function of stimulus duration (B) but did not differ for small and large magnitude digits (C, right). Error bars show *SE*s based on the by-participant analysis (respectively 17 and 28 participants in Experiment 1 and 2).

In Experiment 1, the trimming method described above resulted in the removal of 5.6% of the data. LME modeling showed that reproduced durations increased as a function of stimulus duration (*β* = 0.75, *SE* = 0.09, *t* = 8.35, *p*<.001; see Figure 3A), suggesting that participants were very sensitive to the stimulus duration in their reproductions. Participants also reproduced longer durations if the stimulus duration was presented together with a large magnitude digit than with a small magnitude one (627 vs. 614 ms; *β* = 18.31, *SE* = 8.36, *t* = 2.19, *p* = .030; see Figure 3C, left). The interaction between stimulus duration and numerical magnitude was not significant (*β* = 0.02, *SE* = 0.04, *t* = 0.59, *p* = .568) (The same statistical pattern was obtained when we followed the trimming method in Chang et al.'s study [2] and used ANOVA analyses as they did: there was a significant effect for stimulus duration (*F*(3,48) = 44.69, *p*<.001) and numerical magnitude (*F*(1,16) = 4.85, *p* = .043), but not for the interaction (*F*<1).).

In Experiment 2, 3.3% of the data were removed as outliers. Participants reproduced longer durations for longer stimulus duration (*β* = 0.62, *SE* = 0.05, *t* = 13.81, *p*<.001; see Figure 3B). However, they reproduced similar durations regardless of the magnitude of the digit they saw during reproduction (553 ms for large magnitude digits and 550 ms for small magnitude ones; *β* = 5.33, *SE* = 7.00, *t* = 0.76, *p* = .448; see Figure 3C, right). The interaction was not significant (*β* = 0.002, *SE* = 0.03, *t* = 0.07, *p* = .944) (Again, using the trimming and analyses used by Chang et al. did not alter the statistical pattern: there was a significant effect for stimulus duration (*F*(3,81) = 119.21, *p*<.001) but not for numerical magnitude or the interaction (*F*s<1)).

In Experiment 1, we replicated the finding in Chang et al.'s Experiment 1 that large magnitudes lengthen reproduced durations when they are concurrently presented at the perception stage (i.e., at the time when a stimulus duration is being perceived), but we failed to replicate the finding in their Experiment 2 that large magnitudes shorten reproduced times when they are concurrently presented at the reproduction stage (i.e., at the time when participants produce a new duration to match the memorized stimulus duration). It should be noted that it is possible that our Experiment 2 failed to replicate the result in Chang et al.'s study because participants in our experiment did not pay attention to the digit occurring during reproduction. This is possible since our experiments differed from those in Chang et al.'s study in not having a 1000 ms blank interval between the offset of the stimulus duration and the onset of the reproduction cue (i.e., “NOW”). Possibly, the presence of the interval would direct participants' attention to the screen as they needed to detect the presence of the visual cue before beginning the reproduction. Thus, in Experiment 3, we introduced the 1000 ms blank interval on the basis of Experiment 2; furthermore, to ensure that participants pay attention to the digit, we also introduced a digit reproduction task in which participants press a key in the number pad corresponding to the digit they have seen during time reproduction.

## Experiment 3

### Method

The purpose of Experiment 3 was to test whether the lack of the numerical magnitude effect in Experiment 2 was due to participants not paying attention to the digit presented during reproduction (and also due to the absence of the blank interval between the offset of the green dot and the onset of “NOW”). Thus, the design of Experiment 3 was the same as that of Experiment 2 except that we introduced a 1000 ms blank interval between the offset of the green dot and the onset of “NOW” and participants had to reproduce the digit they saw during time reproduction by pressing the corresponding key on the number pad. That is, in Experiment 3, participants first saw a fixation for 200 ms followed by a blank screen of 800 ms. Then they saw a green dot lasting for one of the four stimulus durations (300, 450, 600, or 700 ms). After a blank screen of 1000 ms, they saw “NOW”. When they held down the key “0”, a digit (1, 2, 8, or 9) appeared and stayed onscreen for the whole reproduction. After the time reproduction, they saw “?” on the screen and pressed a key to reproduce the digit. Thirty-three participants (25 females; mean age: 19.5) from the same population as those in Experiments 1 and 2 were paid 10 Yuan to take part.

### Results and discussion

For the digit reproduction task, participants correctly reproduced the digit in 99.3% of the trials on average (87%–100%), suggesting that they had paid close attention to the digit that appeared during duration reproduction. For the reproductions, we followed the trimming method in Experiments 1 and 2. One participant was removed from further analysis for reproducing durations shorter than 1/4 of the stimulus duration for more than 60% of the trials (his/her inclusion did alter the statistic pattern though). We then further removed trials in which participants did not correctly reproduce the digit and reproductions that were shorter than 1/4 of the stimulus duration or longer than 4 times the stimulus duration, resulting in the removal of 4.9% of the data.

**Figure 4:**
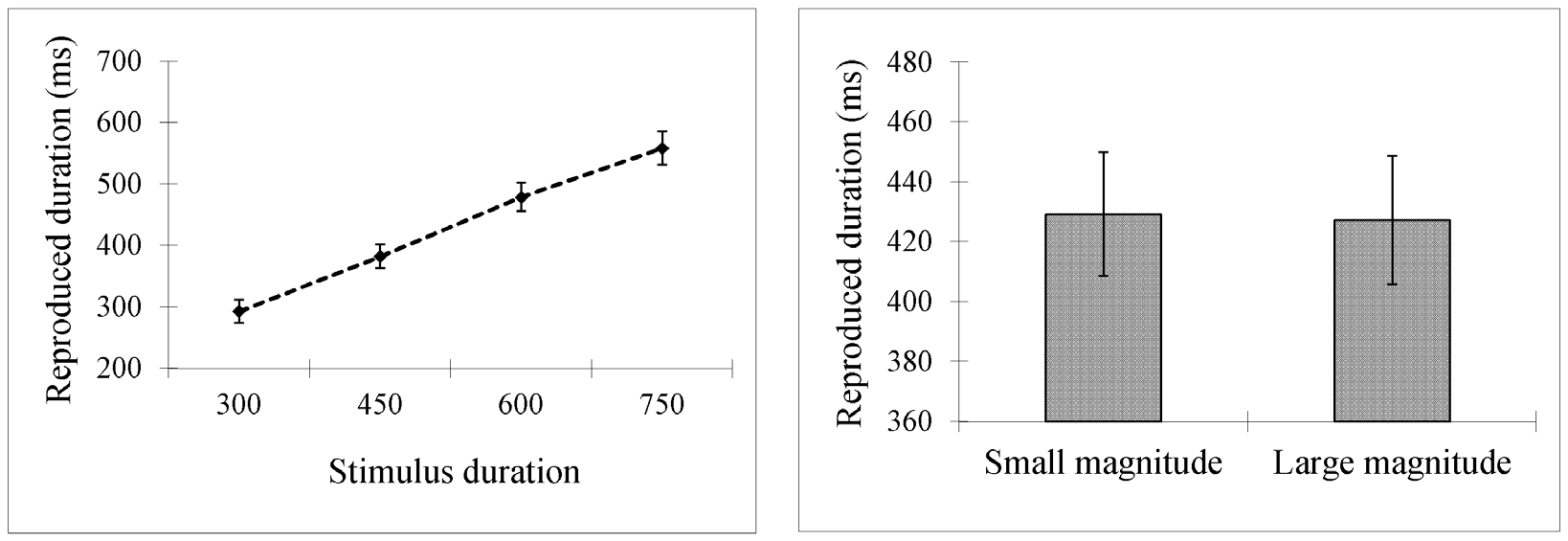
Results for Experiment 3. Reproduced durations increased as a function of stimulus duration (A) but did not differ for small and large magnitude digits (B). Error bars show *SE*s based on the by-participant analysis (33 participants in total).

LME analyses on the remaining reproductions reveal a main effect of stimulus duration, with reproduced durations increasing as a function of stimulus duration (*β* = 0.59, *SE* = 0.04, *t* = 14.08, *p*<.001; see Figure 4A). Numerical magnitude did not produce a significant effect (*β* = 1.94, *SE* = 2.83, *t* = 0.68, *p* = .492), with very similar reproductions for large and small magnitude digits (412 ms vs. 414 ms; see Figure 4B). Numerical magnitude did not significantly interact with stimulus duration (*β* = 0.01, *SE* = 0.02, *t* = 0.75, *p* = .460) (The same statistical pattern was obtained using the methods in Chang et al.: there was a significant main effect for stimulus duration (*F*(3,96) = 126.15, *p*<.001), but not for numerical magnitude (*F* (1,32) = 1.58, *p* = .217). The interaction was not significant (*F*<1).).

As in Experiment 2, in this experiment, participants' reproductions were not influenced by the numerical magnitude of the digit they saw during time reproduction. As we inserted a blank interval of 1000 ms between the offset of the green dot and the onset of “NOW” (as Chang et al. did), the results of this experiment suggest that the lack of the numerical magnitude effect in Experiment 2 was not due to the absence of the blank interval; otherwise we should have observed the effect in this experiment. Furthermore, the results of this experiment also help to rule out the inattention explanation for the lack of the numerical magnitude effect in Experiment 2: in Experiment 3, our analysis focused only on trials in which participants correctly reproduced the digit; still, the magnitude of the digit failed to influence temporal reproductions. In the following experiments, we made further attempts to replicate the findings in Chang et al. [2] by treating numerical magnitude as a continuous variable as it should be, rather than a categorical one.

## Experiments 4a, 4b and 5

These three experiments followed the basic designs in Experiments 1 and 2 but treated numerical magnitude as a continuous variable. We selected 5 digits 1, 3, 5, 7, and 9, and used the same four stimulus durations (300, 450, 600, and 750 ms) used in Experiments 1 and 2. We manipulated numerical magnitude at the perception stage in Experiment 4a, but at the reproduction stage in Experiment 4b and Experiment 5. Experiment 5 increased both participants and items over Experiment 4b as a more powerful attempt to test the effect of numerical magnitude presented at the reproduction stage. If the internal clock speeds up in the presence of a large magnitude digit, we should expect reproduced durations to increase as a function of numerical magnitude presented at the perception stage (Experiment 4a) but to decrease as a function of numerical magnitude presented at the reproduction stage (Experiments 4b and 5). If instead numerical magnitude affects temporal memories, we should only expect the numerical magnitude effect when the digits were presented at the perception stage (Experiment 4a).

### Method

The method was identical to that in Experiments 1 and 2 except for the following changes. Magnitude was manipulated as a continuous variable using five digits: 1, 3, 5, 7 and 9. Crossing the five digits and the four stimulus durations resulted in twenty digit-duration combinations in each block of materials, whose order was randomized. There were six blocks of materials in Experiment 4a and 4b, and 10 blocks in Experiment 5. Twenty new participants (16 females; mean age: 21) from the South China Normal University were paid 10 Yuan to take part in Experiment 4a and 4b. They did Experiment 4a first and after a break of about five minutes they continued with Experiment 4b. Another 26 participants (21 females; mean age: 21.5) were recruited to take part in Experiment 5.

### Results and discussion

**Figure 5:**
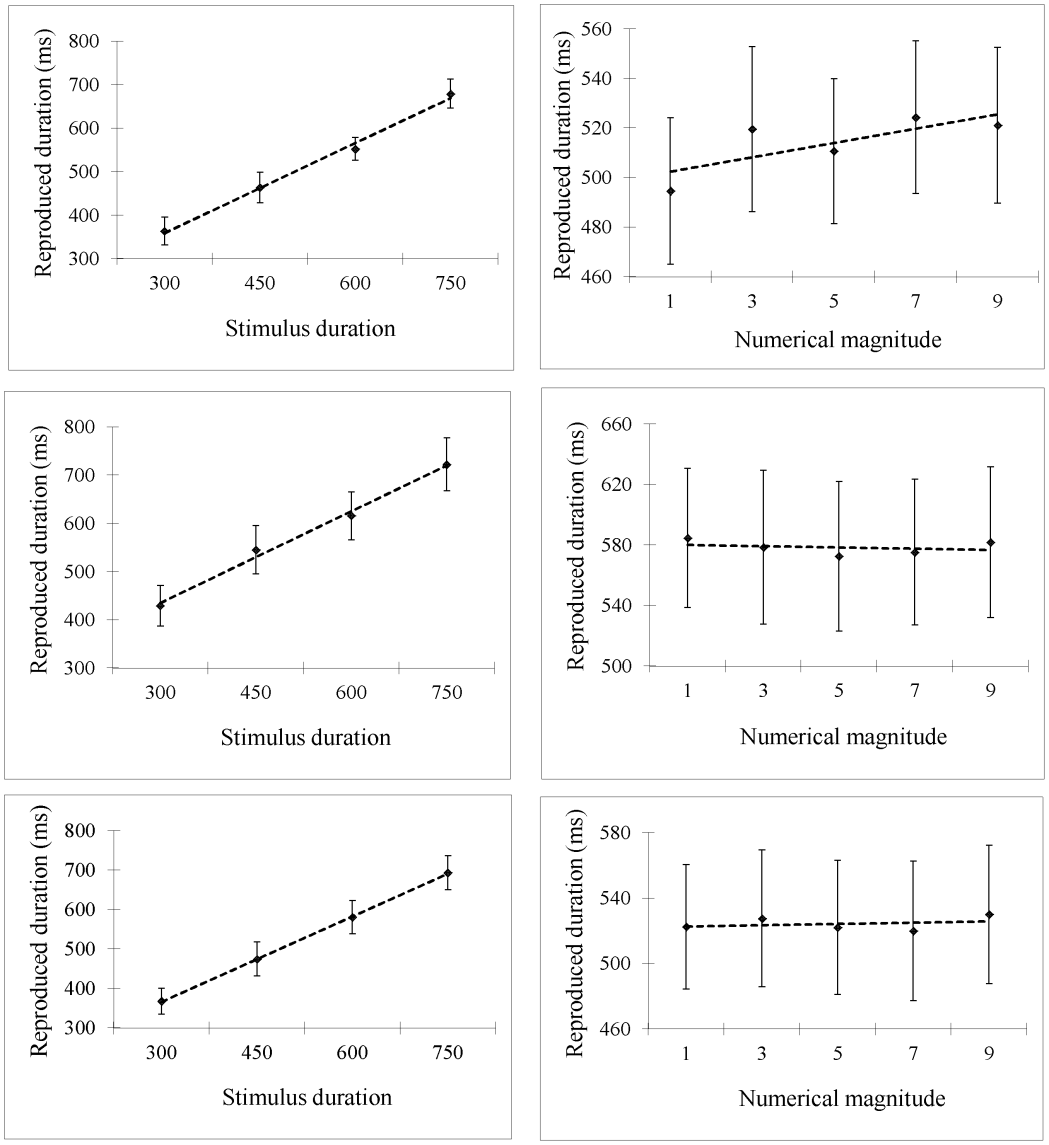
Results for Experiment 4a, 4b, and 5. In Experiment 4a, reproduced durations increased as a function of stimulus duration (A) and as a function of numerical magnitude (B). In both Experiments 4b and 5, reproduced durations increased as a function of stimulus duration (C, E) but not as a function of numerical magnitude (D, F). Error bars show *SE*s based on the by-participant analysis (20 participants in Experiment 4a,b and 26 participants in Experiment 5).

In Experiment 4a, we excluded 2.6% of the data as outliers. LME modelling reveals that reproduced durations increased as a function of stimulus duration (*β* = 0.68, *SE* = 0.06, *t* = 11.64, *p*<.001; see Figure 5A) and as a function of the numerical magnitude of the digits concurrently presented with the stimulus duration at the perception stage (*β* = 2.72, *SE* = 1.28, *t* = 2.12, *p* = .035; see Figure 5B). The interaction did not produce a significant effect (*β* = 0.003, *SE* = 0.007, *t* = 0.32, *p* = .747).

In Experiment 4b, we excluded 1% of the data was outliers. Reproduced durations increased as a function of stimulus duration (*β* = 0.61, *SE* = 0.08, *t* = 8.16, *p*<.001; see Figure 5C) but not as a function of the numerical magnitude of the digits presented at the reproduction stage (*β* = 0.43, *SE* = 1.22, *t* = 0.35, *p* = .726; see Figure 5D). The interaction was not significant (*β* = −0.01, *SE* = 0.007, *t* = 1.38, *p* = .169).

In Experiment 5, we excluded two participants as 80% of their reproduced durations were less than 1/4 of the stimulus durations (their inclusion did alter the statistic pattern though). We then excluded 6% of the remaining data as outliers. The statistical pattern was the same as in Experiment 4b. Reproduced durations increased as a function of stimulus duration (β = 0.68, SE = 0.05, t = 12.80, p<.001; see Figure 5E), but not as a function of the numerical magnitude of the digits presented at the reproduction stage (β = −0.31, SE = 1.35, t = −0.23, p = .821; see Figure 5F). The interaction was not significant (β = −0.004, SE = 0.008, t = −0.54, p = .592).

Experiment 4a presented a more rigorous test of the numerical magnitude effect than Experiment 1 by treating numerical magnitude as a continuous variable. The results corroborate those in Experiment 1: large numerical magnitudes, when presented during the perception stage, lengthened reproduced durations. Experiment 4b corroborates the finding in Experiments 2 and 3, showing that numerical magnitude did not affect time perception when digits were presented at the reproduction stage, whether they were treated as categorical or continuous. Such a finding was further supported by Experiment 5, where more items and more participants were tested to increase power.

## General Discussion

In Experiment 1, we replicated previous findings that a large magnitude digit (8 or 9), relative to a small magnitude one (1 or 2), increased perceived duration when the digit was concurrently presented with the stimulus duration (i.e., at the perception stage) [1], [2]. However, in contradiction with the finding in Chang et al. [2], in Experiment 2, a large magnitude digit did not lead to shorter reproduced durations than a small magnitude digit when digits were presented at the time when participants were reproducing the stimulus duration (i.e., at the reproduction stage). Such a null effect was also observed in Experiment 3, where we had participants explicitly attend to the digit presented during time reproduction by asking them to later reproduce the digit. The finding that numerical magnitude affects perceived duration when digits are presented at the perception stage but not at the reproduction stage is further confirmed when we manipulated numerical magnitude as a continuous variable: reproduced durations increased as a function of numerical magnitude when digits were presented at the perception stage (Experiment 4a) but not at the reproduction stage (Experiments 4b and 5).

The lack of the numerical effect in experiments where the digit was presented during reproduction was unlikely due to inattention to the digit. Note that Experiment 3 demonstrated that reproductions from trials where the digit was later correctly reproduced were insensitive to the magnitude of the digit presented during time reproduction. Also, the null effect of numerical magnitude in Experiment 2 could not be attributed to the absence of the 1000 ms blank interval (which was present in Chang et al.'s Experiment 2), as the null effect was further confirmed in Experiment 3 with the interval installed. Finally, the lack of the numerical magnitude effect was unlikely due to a lack power in our experiments. First, our Experiment 2 had more participants than the corresponding experiment in Chang et al. (28 vs. 24 participants). Second, the experiments in which we failed to observe the numerical magnitude effect had more observations than the ones where we did observe the effect (i.e., more participants in Experiments 2 and 3 than Experiment 1; and more participants and items in Experiment 5 than Experiment 4a).

Though it might not be very convincing to draw a conclusion from a null effect, the repetitive replication failures urged us to conclude that numerical magnitude of digits affects perceived time when digits are concurrently presented during the perception of the stimulus duration but not during the reproduction of the stimulus duration. These findings suggest that numerical magnitude does not bias the clock speed during time encoding (at either the time perception or reproduction stage), as otherwise we should have observed reproduced durations decreasing as a function of the numerical magnitude presented at the reproduction stage. Furthermore, it has been suggested that successful manipulation of the clock speed should result in a slope change in the effect over stimulus duration [32]–[34]. In the current context, if the clock speed is accelerated by a large numerical magnitude, we should expect the difference in reproduced durations between a large and a small numerical magnitude to increase as a function of the stimulus duration (in other words, we should have observed a statistically significant interaction between numerical magnitude and stimulus duration; see [32–24]). However, in all the experiments, numerical magnitude never produced such a slope effect across the stimulus duration, a result that further fortifies the conclusion that numerical magnitude does not affect the “ticking” rate of the internal clock.

Instead, the findings from the experiments are consistent with those observed by Cai and Connell (unpublished data) regarding the effect of spatial distance on time perception: people reproduced longer durations for a long-distance line than for a short-distance line presented at the perception stage but not at the reproduction stage. If we assume that both numerical magnitude and spatial distance are represented as mental magnitudes [9], [10], the findings in the current study and in Cai and Connell (unpublished data) converge to suggest that non-temporal magnitude information interacts with a perceived duration when they concurrently reside in memory. Note that even in tasks where participants do not have to explicitly remember the non-temporal dimension for a later task (as in our Experiment 1), attending to a digit will suffice to leave a representation of that digit in memory. For instance, in a task such as the one in Experiment 1, we would not have much difficulty recalling the digit we saw during perception when unexpectedly asked to do so, which indicates that we retain some memory of the digits. In fact, it was shown that concurrent spatial distance affected time perception to the same extent whether participants had to explicitly remember the distance or to simply attend to it [7]. Thus, under the above account, when presented at the perception stage, a digit has the opportunity to co-exist in memory with a perceived duration and to bias the noisy magnitude representation of the duration, with a large magnitude digit lengthening the temporal memory relative to a small magnitude digit, as observed in Experiment 1 and 4a. However, when the digit is presented at the reproduction stage, the digit should not affect the memory of a perceived duration as the memory has been retrieved when the digit is presented. Such an account thus explains the lack of the numerical magnitude effect when digits are presented during reproduction (Experiments 2, 3, 4b, and 5).

The account complements the magnitude theory on the perception of quantifiable dimensions such as time, space, and number. According to the theory, these dimensions are collectively processed in the parietal cortex [10], [11], which gives rise to the interference among the different dimensions. The theory, however, is underspecified as to whether the interference arises during the actual perception of the dimensions or after the perceived information is stored in memory. Our findings provide support for the latter interpretation of the magnitude theory. Furthermore, our interpretation of the magnitude theory will help to bring two separate branches of time perception research: time representation and time processing. More specifically, we localize the effect of magnitude information of time in the memory component of the internal clock model; that is, time is encoded by a clock-like device (e.g., as pulses) and then stored in memory as approximate and noisy mental magnitudes. Such an integrated framework, on the one hand, enables the internal clock model to account for the interaction between time and other magnitude dimensions, and on the other hand, provides an information-processing framework for the magnitude theory of time representation.
