# Correction: Numerical Magnitude Affects Temporal Memories but Not Time Encoding

**DOI:** 10.1371/journal.pone.0096885

**Published:** 2014-05-01

**Authors:** The PLOS ONE Staff

The corresponding author’s email address is incorrect. The correct email address is: zhenguangcai@gmail.com

Figure 1 is incorrect. The authors have provided a corrected version here.

**Figure 1:**
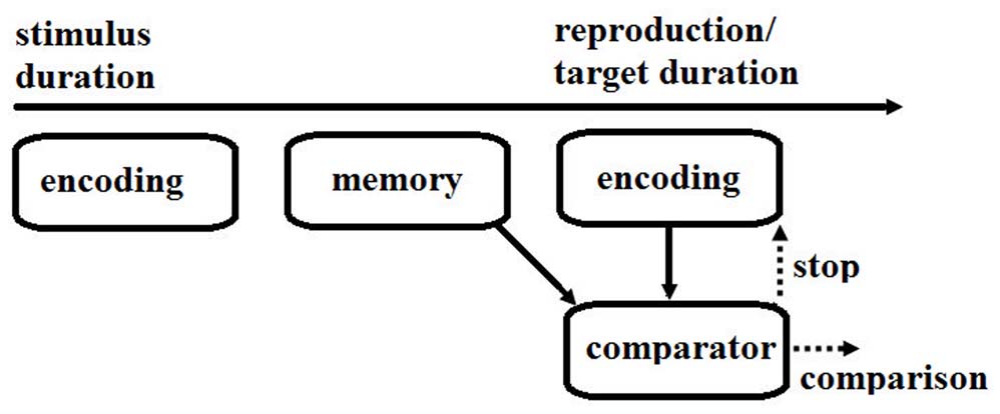
Cognitive processes involved in time perception. First, a stimulus duration is encoded and then kept in memory. Next, the comparator retrieves the remembered duration with which the newly encoded duration (i.e., the duration being reproduced in a reproduction task or a target duration in a comparison task) is compared. The comparator stops the reproduction when the reproduced duration is similar enough to the remembered duration, or makes a comparison judgment based on the relative amounts of time between the remembered duration and the target duration in a comparison task.
